# INFUSE: Rationale and design of a multi-center, open label, collaborative study to treat HRS-AKI with continuous terlipressin infusion

**DOI:** 10.1016/j.conctc.2023.101211

**Published:** 2023-10-05

**Authors:** Ethan Weinberg, Suditi Rahematpura, Stevan A. Gonzalez, Manhal J. Izzy, Douglas A. Simonetto, R. Todd Frederick, Raymond A. Rubin, Jade Ikahihifo-Bender, Maggie Harte, Grace Kim-Lee, Sherry Witkiewicz, William Tobin, Khurram Jamil, Zachary Fricker, K. Rajender Reddy

**Affiliations:** a Division of Gastroenterology and Hepatology, University of Pennsylvania Perelman School of Medicine, Philadelphia, PA, USA; b Division of Hepatology, Simmons Transplant Institute, Baylor Scott and White All Saints Medical Center, Fort Worth, TX, USA; c Department of Gastroenterology, Hepatology, and Nutrition, Vanderbilt University Medical Center, Nashville, TN, USA; d Division of Gastroenterology and Hepatology, Mayo Clinic, Rochester, MN, USA; e Department of Hepatology and Liver Transplantation, California Pacific Medical Center, San Francisco, CA, USA; f Department of Transplantation, Piedmont Transplant Institute, Atlanta, GA, USA; g International Healthcare, LLC, Norwalk, CT, USA; h Mallinckrodt Ltd, Scientific Affairs, Hampton, NJ, USA; i Division of Gastroenterology, Hepatology, and Nutrition, Beth Israel Deaconess Medical Center, Harvard Medical School, Boston, MA, USA

**Keywords:** Decompensated cirrhosis, Portal hypertension, Ascites, Hepatorenal syndrome, Liver transplant

## Abstract

**Background:**

Hepatorenal syndrome-acute kidney injury (HRS-AKI) carries significant morbidity and mortality among those with end-stage liver disease. Bolus terlipressin for treatment of HRS-AKI received FDA approval in September 2022. US implementation of terlipressin, however, is hindered by the paucity of local data on the optimal patient population and administration mode, as well as the effect on transplant priority. The INFUSE study is designed to evaluate the use of continuous terlipressin infusion among transplant candidates with advanced liver disease and HRS-AKI.

**Methods:**

Fifty prospective patients with HRS-AKI will receive a single bolus of terlipressin 0.5 mg followed by continuous infusions of terlipressin from 2 to 8 mg/day for up to 14 days. The cohort will be enriched with those listed, in evaluation, or eligible for liver transplantation, while those with ACLF grade 3, MELD ≥35, and serum creatinine >5.0 mg/dL will be excluded. Fifty patients who received midodrine plus octreotide or norepinephrine for HRS-AKI will serve as a retrospective comparator cohort.

**Conclusion:**

The INFUSE study aims to assess the safety and efficacy of continuous terlipressin infusion among largely transplant-eligible patients with HRS-AKI, and to provide US-based data on transplant outcomes. This novel study design simultaneously mitigates terlipressin adverse events while providing renal benefits to patients, thus addressing the unmet medical need of those with HRS-AKI who have limited treatment options and are awaiting liver transplantation in the US.

## Abbreviations

HRS-AKI: hepatorenal syndrome-acute kidney injury
SCr: serum creatinine
ICA: International Club of Ascites
KDIGO: Kidney Disease: Improving Global Outcomes
M&O: midodrine and octreotide
RCT: randomized controlled trial
ACLF: acute-on-chronic liver failure
RRT: renal replacement therapy
AEs: adverse events
MELD: model for end-stage liver disease
INFUSE: Continuous Infusion of Terlipressin for Subjects with HRS-AKI on the Liver Transplant Waiting List
ICH: International Council for Harmonisation of Technical Requirements for Pharmaceuticals for Human Use
EOT: end of treatment
SAEs: serious adverse events

## 1 Introduction

Hepatorenal Syndrome-Acute Kidney Injury (HRS-AKI) is a form of renal dysfunction seen in patients with advanced liver disease and is associated with significant morbidity and mortality [1]. Approximately 4% of patients hospitalized with decompensated cirrhosis develop HRS-AKI on that admission, with subsequent probability of disease occurrence estimated at 18% and 39% over 1-year and 5-years, respectively [2]. The nomenclature and diagnostic criteria of HRS have undergone significant revision over time. HRS was previously categorized into HRS type 1, defined as a doubling in serum creatinine (SCr) to > 2.5 mg/dL within 2 weeks, or HRS type 2, defined as a slower, more chronic increase in SCr [3,4]. In 2015, the International Club of Ascites (ICA) proposed new criteria for HRS in order to apply Kidney Disease: Improving Global Outcomes (KDIGO) AKI guidelines to patients with cirrhosis, accounting for short-term, dynamic changes in SCr and allow for earlier intervention [5,6]. HRS type 1 was renamed HRS-AKI and aligned with the KDIGO AKI definition (i.e. ≥ 0.3 mg/dL increase in SCr within 48 hours or ≥1.5-fold increase in SCr within 7 days, Table 1), and HRS type 2 was renamed HRS-CKD to indicate its chronic nature [[6], [7], [8], [9]].

**Table 1:** HRS-AKI Diagnostic Criteria based on KDIGO AKI guidelines [5,6,9].

Key Diagnostic Criteria for HRS-AKI [5,6,9]
• Cirrhosis and ascites • AKI defined as ≥ 0.3 mg/dL increase in SCr within 48 hours or ≥ 1.5-fold increase in SCr within 7 days o Stage 1a: ≥0.3 mg/dL increase or ≥ 1.5-fold to 2-fold above baseline o Stage 1 b: ≥0.3 mg/dL increase or ≥ 1.5-fold to 2-fold above baseline to SCr ≥1.5 mg/dL o Stage 2: > 2-fold to 3-fold above baseline o Stage 3: > 3-fold above baseline, or SCr ≥4 mg/dL with an acute increase ≥0.3 mg/dL or initiation of RRT • No improvement in SCr after ≥48 hours of diuretic withdrawal and volume expansion with albumin at a dose of 1 g/kg/day with a max of 100 g/day • Absence of hypovolemic shock or infection requiring vasoactive drugs to support blood pressure • No current or recent use of nephrotoxic drugs • Proteinuria <500 mg/day and hematuria <50 red blood cells/high-power field

Prior to 2022 in the United States, the standard of care for HRS-AKI was off-label treatment with midodrine and octreotide (M&O) plus albumin. This was in contradistinction to global guidelines and studies, where the vasopressin analog terlipressin has been the preferred and more efficacious treatment option for HRS-AKI [10,11], and norepinephrine as an alternative [[12], [13], [14]]. A 2015 multi-center randomized controlled trial (RCT) in Italy noted terlipressin plus albumin to be superior to M&O plus albumin in the treatment of HRS [10]. While some studies have shown that M&O was associated with improved liver-transplant free survival in patients with HRS, these studies were non-randomized and retrospective in nature [[15], [16], [17]]. Further, a 2017 meta-analysis of 13 worldwide RCTs found moderate-quality evidence for terlipressin and low-quality evidence for norepinephrine as more efficacious treatments than M&O [18]. Norepinephrine demonstrated similar efficacy to terlipressin in several small studies with 20–46 patients, but a 2020 RCT in India among 120 patients with acute-on-chronic liver failure (ACLF) found norepinephrine to be less efficacious than terlipressin and associated with higher rates of renal replacement therapy (RRT) and mortality [[19], [20], [21], [22], [23]]. Norepinephrine also poses clinical challenges with central line placement and ICU admissions [20,24].

While the bulk of terlipressin studies have been non-US based, several clinical studies in North America have also demonstrated greater rate of HRS reversal and renal improvement with bolus terlipressin compared to placebo [[25], [26], [27]]. The most comprehensive of these is the CONFIRM study in which rates of HRS reversal were nearly double among patients treated with bolus terlipressin compared to those who received placebo [27]. One concern has been that patients who received terlipressin in CONFIRM had a marginally lower rate of liver transplantation [27]. The CONFIRM study also noted that bolus terlipressin was associated with a higher rate of adverse events (AEs) than placebo. Among severely-ill patients with advanced liver disease, i.e. Model for End-Stage Liver Disease (MELD) score greater than 35 or ACLF grade 3, terlipressin was associated with a higher incidence of respiratory failure and greater respiratory-related mortality within 90 days [27]. Of note, a 2016 multi-center RCT in Italy found that continuous intravenous infusion of terlipressin was better tolerated and more effective with a lower total daily doses than bolus terlipressin, with significantly more AEs in bolus cohort vs continuous infusion group even with patients having relatively lower MELD scores compared to CONFIRM. In the Cavallin et al., 2016 study, baseline MELD scores (infusion cohort; bolus cohort): 29.26 ± 7.76; 29.81 ± 6.40, were lower compared with CONFIRM cohorts where baseline MELD scores were (bolus cohort; placebo cohort): 32.7 ± 6.6; 33.1 ± 6.2 [27,28].

HRS-AKI had no approved treatment in the US prior to FDA-approval of bolus-injection terlipressin in September 2022 [29]. This is a major step in addressing the unmet need associated with HRS-AKI; yet the lack of robust, local data regarding the limitations of terlipressin continues to hinder its implementation in the US. Thus, the impact of HRS-AKI reversal in transplant candidates and the mitigation of AEs with a continuous terlipressin infusion strategy as opposed to a bolus strategy are research areas of great interest. The present study aims to assess the safety and efficacy of continuous terlipressin infusion for HRS-AKI in a cohort enriched with patients eligible, in evaluation, or listed for liver transplant.

## 2 Patients and methods

The Continuous INfusion oF Terlipressin for SUbjects with HRS-AKI on the LivEr Transplant Waiting List (INFUSE) study is a multi-center study conducted across the US. Participating centers include: University of Pennsylvania, Vanderbilt University, Beth Israel Deaconess Medical Center, Mayo Clinic, Baylor Scott & White Medical Center, Piedmont Atlanta Hospital and California Pacific Medical Center.

This study is conducted in accordance with the elements of Good Clinical Practice, as defined by the International Council for Harmonisation of Technical Requirements for Pharmaceuticals for Human Use (ICH) and all local and national regulations. Prior to the initiation of study-specific procedures, study candidates at each center voluntarily sign informed consent forms consistent with the ethical guidelines of the Declaration of Helsinki and approved by each centers' Institutional Review Board. The study has been registered on ClinicalTrials.gov (NCT04460560) and has an IND (146962).

The INFUSE study has been designed to address the following aims:

Aim 1: To assess the safety and efficacy of continuous terlipressin infusions in adults with HRS-AKI who are on the transplant waitlist or who are transplant eligible with anticipation of being placed on the liver transplant waitlist. Aim 2: To assess the safety and efficacy of continuous terlipressin infusions in adults with HRS-AKI who are not eligible for liver transplantation.

### 2.1 Trial participants

This study has prospective and retrospective cohorts to accomplish these aims. In the prospective terlipressin cohort, 50 patients across the study sites will be treated. A retrospective comparator cohort includes 50 historical patients across study sites who received at least 48 hours of M&O or norepinephrine for HRS-AKI and would have otherwise met INFUSE study inclusion/exclusion criteria (Table 2).

**Table 2:** Inclusion and exclusion criteria for open label terlipressin trial for hrs-aki

Key Inclusion Criteria
• At least 18 years of age • Cirrhosis and ascites • No sustained improvement in renal function (less than 20% decrease in SCr levels) at least 48 hours after diuretic withdrawal and plasma volume expansion with albumin (given daily for 2 days–48 hours from the 1st dose) OR if SCr improves by ≥ 20% but plateaus (≤10% fluctuation in SCr) and remains above 1.5 mg/dL for at least another 48 hours and there are no features of tubular necrosis • AKI stage 1b or above and increase to SCr levels of ≥1.5 mg/dL at the time of initiating treatment o Baseline SCr is defined as the most recent, lowest SCr in the last 6 months before current admission • On liver transplant waitlist or transplant eligible with anticipation of being placed on the liver transplant waitlist OR not on transplant waitlist or ineligible for transplant (max 25 subjects)
**Key Exclusion Criteria**
• SCr >5.0 mg/dL^a^ • MELD score ≥35 • ACLF grade 3 (according to CLIF Consortium grading system) • Uncontrolled sepsis and/or bacterial infection • Shock • Current or recent (within 4 weeks) treatment with or exposure to nephrotoxic agents • Estimated life expectancy of <7 days • Advanced hepatocellular carcinoma with estimated life expectancy of <6 months • Superimposed acute liver injury due to drug, dietary supplements, herbal preparations, viral hepatitis or toxins, with the exception of acute alcoholic hepatitis • Evidence of obstructive uropathy or parenchymal renal disease • Evidence of tubular necrosis and/or intrinsic renal disease • Severe cardiovascular disease • Current or recent (within 4 weeks) RRT or anticipation of RRT within 3 days on enrollment • TIPS within 30 days of starting study drug • Prospective terlipressin cohort: all vasopressors must be stopped prior to terlipressin treatment

a Subjects with SCr levels greater than 5.0 mg/dL may be enrolled with Sponsor approval.

### 2.2 Drug stability and bacteriology

In-use stability testing was conducted by Pharmaceutical Development Services at Patheon; microbiology sterility testing was conducted by the Investigational Drug Service at the Perelman School of Medicine. Reconstituted terlipressin (0.85 mg terlipressin free base in 0.9% sodium chloride solution) was tested for appearance, pH, terlipressin assay, impurities, aerobic bacteria, anaerobic bacteria, and pyrogen at 2 °C to 8 °C (refrigerated conditions) and 25 °C (room temperature) for up to 72 hours. The solution passed all parameters at both refrigerated conditions and room temperature up to 72 hours. A previous study conducted by Bui et al. demonstrated sterility for up to 7 days at 2 °C to 8 °C (refrigerated conditions) and for 24 hours at 22.5 °C (room temperature) [30].

Terlipressin is provided to sites in single-use, sterile 6-mL vials containing 1 mg of lyophilized terlipressin acetate, which is equivalent to 0.85 mg of terlipressin free base. The drug is stored in a secure location at 2 °C to 8 °C until reconstitution. Reconstituted bolus and infusion bags may be stored up to 48 hours at 2 °C to 8 °C or up to 24 hours at 25 °C.

### 2.3 Drug administration and dose escalation (Fig. 1)

Prospective patients are given an initial bolus dose of terlipressin prior to continuous infusion to achieve a therapeutic level earlier than continuous infusion alone. The bolus dose is prepared by reconstituting 1 vial of 1 mg terlipressin acetate with 5 mL of sterile 0.9% sodium chloride solution and given over a 1-minute push. A 0.5 mg bolus dose is utilized to mitigate potential side effects, such as diarrhea and abdominal pain, observed when patients in the CONFIRM study received 1.0 mg bolus doses of terlipressin every 6 hours [27].

**Fig. 1:**
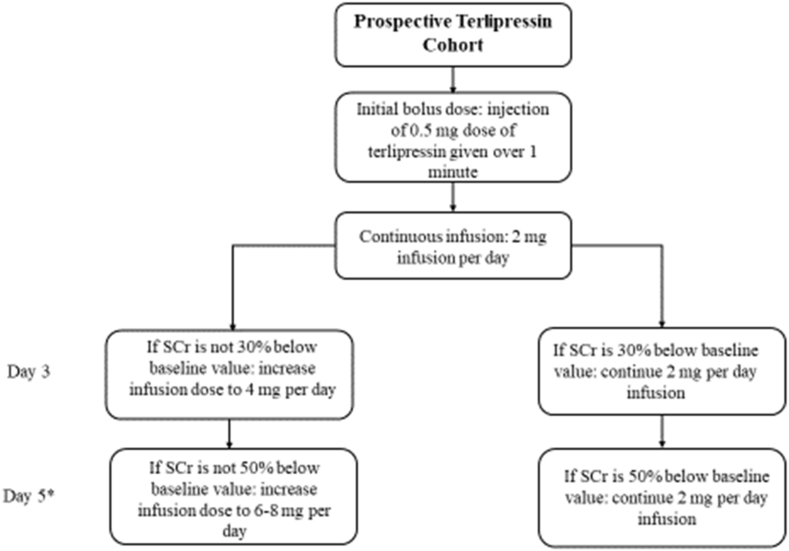
Terlipressin Dose Escalation for Prospective Terlipressin Cohort * If 8 mg maxed dose not reached on Day 5, dose may be increased up to 8 mg on Days 6–14.

Immediately following this initial 0.5 mg bolus, the continuous infusion of terlipressin begins with an initial dose of 2 mg of terlipressin infused with a pump over 24 hours. Dosing starts at 2 mg/day and increases up to 8 mg/day based on SCr response and tolerability as follows: increase to 4 mg/day if SCr does not decrease at least 30% from baseline by day 3; increase to 6–8 mg/day, per PI discretion, if SCr does not decrease at least 50% from baseline by day 5 (Fig. 1). The IV terlipressin solution bag is changed every 24 hours. In the event of an adverse reaction, stopping and restarting of terlipressin is left up to the discretion of the investigator. For example, adverse GI side effects do not necessarily warrant interrupting or permanently discontinuing the infusion, while an ischemic event would intuitively lead to permanent discontinuation. If the infusion is paused for 4 hours or more, a 0.5 mg bolus is to be administered prior to restarting the infusion due to the 50-min half-life of terlipressin. Terlipressin acetate is diluted with sterile 0.9% sodium chloride to prepare the various continuous infusion doses then infused at the corresponding hourly rate (Table 3).

**Table 3:** Dilution and hourly infusion rate of continuous terlipressin doses.

Daily Terlipressin Acetate Dose (mg)	Volume of 0.9% Sodium Chloride for Dilution (mL)	Hourly Rate of Fluid Infusion (mL/hr)	Hourly Rate of Terlipressin Infusion (mg/hr)
2	100	4.17	0.083
4	100	4.17	0.167
6	150	6.25	0.250
8	200	8.33	0.333

### 2.4 Schedule of events

Patients are screened to ensure that they meet the inclusion and exclusion criteria (Table 2). They are then monitored for up to 14 days of terlipressin treatment, with the main objective being daily SCr level monitoring to track how effectively terlipressin improves renal function. If reversal of HRS-AKI or maximum anticipated effect is achieved prior to the end of 14 days of treatment, terlipressin could be stopped per PI discretion. There is a guidance provided to the clinical sites where daily albumin dosing while on terlipressin would best be based on serum albumin levels (Table 4), while patients are monitored for clinical signs of volume overload. Patients are followed through the end of treatment (EOT) and up to 365 days after treatment end for survival and transplant outcomes (Table 5). Patients who return by day 90 of follow-up with similar symptoms and again meet eligibility criteria are retreated. Those who receive liver transplants within day 365 of follow-up are followed for 1-year post transplant (Table 6).

**Table 4:** Daily albumin dosing guidance.

	Serum albumin (g/dL)	Albumin dose (g)/day
If NO concern for respiratory volume overload	<3.5	25–50
	3.5–3.7	12.5–25
	≥3.8	none
If concerned for respiratory volume overload (tachypnea, clinical examination, chest X-Ray, supplemental oxygen requirement)	Hold albumin

**Table 5:** Key schedule of events and data collection.

Assessment	Screening	Baseline	Treatment - 14 days								End of Treatment	Follow Up (7 ± 2 days, 14 ± 3 days, 30 ± 7 days, 90 ± 7 days, 180 ± 14 days, 365 ± 14 days)
			1	2	3	4	5	6	7	8–14		
Inclusion and exclusion criteria	x	x										
Screening Evaluation/Consent	x											
ECG & Echocardiogram	o	o										
Childs-Pugh Score		x										
Study Drug Dosing			x	x	x	x	x	x	x	x		
SCr	x	x	x	x	x	x	x	x	x	x	x	x
BUN	o	o	o	o	o	o	o	o	o	o	o	o
eGFR	o	o	o	o	o	o	o	o	o	o	o	o
24 hour Urine Output Volume	x	x	x	x	x	x	x	x	x	x		x^a^
Urine Biomarker Sample		*				*					*	
Blood Biomarker Sample		*				*					*	
Cystatin C		*				*					*	
ALT, AST, ALP, protein, albumin, bilirubin		x	x		x				x		x	x
Serum glucose, calcium, magnesium		x	x		x				x		x	
INR		x	x		x				x		x	x
CBC (neutrophils, lymphocytes, total protein and magnesium)		o	o		o				o		o	
Albumin Administration (if given)	x	x	x	x	x	x	x	x	x	x	x	
Encephalopathy Score		x	x	x	x	x	x	x	x	x		
Serum Electrolytes		x	x	x	x	x	x	x	x	x	x	
Vital Signs		x	x	x	x	x	x	x	x	x	x	
Concomitant medications (blood products and IV fluids)			o	o	o	o	o	o	o	o		
Adverse events			Monitor and record through study period up to 30 days post-treatment									
Renal replacement therapy, TIPS, transplant status and survival status			Monitor and record through study period and follow-up period									
Paracentesis events			Record all events until drug treatment is discontinued									

**x**: mandatory.

**o**: if completed to the standard of care, input.

*: optional.

a Only taken at 7 ± 2 days follow-up time point.

**Table 6:** Schedule of events post liver transplantation.

Assessment	Day of Liver Transplant	Follow-up Period (Days from Day of Liver Transplant)						
		7 ± 2 days	14 ± 3 days	30 ± 7 days	90 ± 7 days	180 ± 14 days	365 ± 14 days	Last known follow-up date
Record events of RRT, TIPS and mortality	x	x	x	x	x	x	x	x
Record all paracentesis	x	x	x	x	x	x	x	x
SCr (BUN & eGFR if taken)	x	x	x	x	x	x	x	x
aMELD, MELD-Na	x							
Dialysis				x	x	x	x	x
Incidence of Kidney Transplant	x	x	x	x	x	x	x	x

a While MELD 3.0 was not in place at time of study design, we will endeavor to look at MELD 3.0 at study completion.

### 2.5 Rationale for study controls

The CONFIRM findings show that terlipressin provides a significant therapeutic advantage over placebo treatment for HRS-AKI [27]. Studies of Cavallin et al. previously demonstrated that terlipressin treatment is more effective than treatment with M&O [10], and that continuous infusions of terlipressin may be better tolerated than intravenous bolus doses [28]. Since the benefits of terlipressin therapy have been sufficiently demonstrated, the present study uses historical controls to compare the effects of continuous terlipressin therapy to the current US standard of care, namely vasopressors and albumin. For this same reason, the INFUSE study is also open-label and guarantees all prospective patients access to terlipressin.

### 2.6 Endpoints

The primary efficacy assessment of this study is improvement of renal function in the prospective terlipressin cohort via repeated measures analysis of daily SCr levels from day 1 through EOT. Comparison of these measures to the daily SCr levels observed in the retrospective M&O/norepinephrine cohort will be a secondary efficacy endpoint. Complete response to treatment is defined as ≥ 30% decrease in SCr with EOT SCr ≤1.5 mg/dL. Partial response to treatment is ≥ 30% decrease in SCr with EOT SCr >1.5 mg/dL. Non-response is <30% decrease in SCr.

This study has seven additional secondary endpoints which will be assessed for the prospective terlipressin cohort and in comparison to the retrospective M&O/norepinephrine cohort. These are as follows: (1) need for RRT, (2) survival, (3) transplant, and (4) SCr by days 30 and 90 of follow-up; (5) need for RRT and SCr by days 30, 90, 180, and 365 after liver transplant; (6) incidence of kidney transplant by day 365 after liver transplant; and (7) a descriptive analysis of number of simultaneous liver kidney transplants and episodes of graft rejection by days 90 and 365 after liver transplant.

For the prospective terlipressin cohort, safety review will include: serious adverse events (SAEs) of interest (i.e. ischemia and respiratory failure) and unanticipated AEs while on treatment, and mortality up to 365 days post-treatment or 365 days post-transplant. While mortality will be assessed in the retrospective M&O/norepinephrine cohort, SAEs or AEs will not be captured.

### 2.7 Statistical analysis

For the primary outcome of efficacy of continuous terlipressin infusion for HRS-AKI in the prospective cohort, statistics will be descriptive in nature. For secondary outcomes, including renal outcomes (e.g. EOT and follow-up SCr, need for RRT), survival, and liver transplantation, the prospective terlipressin cohort will be compared to the retrospective M&O/norepinephrine cohort. Chi-squared test or Fisher's exact test will be used for comparing frequency of categorical variables. Non-parametric tests (e.g. Wilcoxon Rank-Sum, Kruskal-Wallis) will be used to compare continuous variables. Competing risk analysis will be performed to assess the rate of survival, RRT, and liver transplantation. Adjustment for covariates will be made using Gray's test for subdistribution hazards.

## 3 Discussion

### 3.1 Patient population

In the CONFIRM study, severely ill patients with advanced liver disease, defined as MELD score >35 or ACLF grade 3, had higher rates of SAEs, including respiratory failure, hepatobiliary disorders, gastrointestinal disorders and infections, and a higher mortality rate [27]. A post-hoc analysis of CONFIRM found ACLF grade 3 compared to grade 1–2 to be a significant predictor of respiratory failure and associated with decreased survival among patients treated with terlipressin [31]. While baseline SCr ≥7 mg/dL was a major exclusion criterion in CONFIRM, post-hoc analyses indicate an association between baseline SCr ≥5 mg/dL and low incidence of HRS reversal by day 14 of treatment [32]. MELD ≥35, ACLF grade 3 and SCr ≥5 mg/dL have been identified as poor prognostic factors for HRS-AKI treatment with terlipressin and as such were excluded from the present study.

### 3.2 Mode of terlipressin infusion

Previous studies investigating terlipressin therapy for HRS-AKI have largely examined bolus terlipressin administration [[25], [26], [27],^33^]. A 2016 study from Cavallin et al. conducted in Italy compared continuous infusions of terlipressin to intravenous boluses and found that, while the response rate was similar between the two routes of administration, continuous infusion was associated with a lower rate of adverse events [28]. Escorsell et al. noted that continuous terlipressin infusions may be more effective than bolus doses in the setting of portal hypertension because bolus terlipressin maintains an effect on portal pressure for less than 4 hours [34]. Given the standard of bolus administration every 6 hours, this leaves a potential gap of approximately 8 hours during which terlipressin is ineffective. Several other non-US studies have also found continuous terlipressin infusion to be convenient, cost effective, safe and efficient at lower daily doses than bolus dosing and in both outpatient and home settings [[35], [36], [37], [38], [39], [40], [41]].

The INFUSE study sets an international paradigm in the US by utilizing continuous terlipressin therapy with bolus doses only given at the start of treatment or to restart treatment after an extended pause. INFUSE is also open label to provide a safe and efficacious therapy, which is typically inaccessible in the US, to all prospective patients who have HRS-AKI and who may be awaiting transplants. All controls utilized in this study are retrospective historical patients, while a prospectively randomized trial would have been ideal. Such a design was however felt to be unethical with the impending approval of terlipressin in the US at a time the INFUSE study was designed.

### 3.3 Transplant priority

The MELD/MELD-Na score is the current metric used to assign transplant priority to patients with end-stage liver disease, where higher values indicate higher transplant priority. A principal concern is the favorable effect of successful terlipressin therapy on MELD score, due to improvement in SCr and hyponatremia, and a subsequent decrease in transplant waitlist priority [42]. However, lower transplant rates may be countered by post-transplant benefits of renal protection and a decrease in the need for RRT as well the subsequent need for renal transplantation. For example, while the proportion of CONFIRM patients who underwent liver transplantation by day 90 was slightly lower among those receiving terlipressin than those receiving placebo, post-hoc analyses indicate a decreased need for pre- and post-transplant RRT and greater RRT-free survival up to 90 days post-transplant in those receiving terlipressin [27,43,44]. This paradox was also seen among patients in Italy who received terlipressin for HRS-AKI before liver transplantation; responders had longer wait times and lower MELD scores at transplantation yet better 30-day transplant-free survival and reduced need for RRT post-transplant [45]. The present study aims to address this paradox by evaluating the rate of liver transplantation in the prospective cohort compared to the retrospective one, as well as the need for simultaneous liver kidney transplants and post-transplant RRT. US-based data on transplant outcomes after terlipressin treatment is of vital interest.

## 4 Conclusion

It has been firmly established through studies conducted within and outside the United States that terlipressin for HRS-AKI is a more efficacious therapy than other vasopressor and albumin strategies [10,[25], [26], [27]]. However, there is no US study examining continuous intravenous infusions of terlipressin, which have been shown to mitigate adverse complications [28], and the interaction between continuous terlipressin therapy and transplant eligibility and priority [46]. Further, there is the question of whether terlipressin treatment in potential transplant recipients decreases the RRT rate both pre- and post-transplant, thus mitigating the adverse outcomes associated with chronic kidney disease as well as reducing the need for renal transplantation.

The INFUSE study applies a global paradigm to the US with its novel study design, namely the first use of continuous terlipressin infusion among largely transplant-eligible patients with HRS-AKI in the US, and its expectation of better tolerability. Based on prior experience, the study has well refined inclusion/exclusion criteria where those at high risk for AEs and those with low likelihood of response, such as patients with MELD ≥35, ACLF Grade 3 or SCr >5, are excluded. The follow-up period, unlike in the Phase III CONFIRM trial, will be for up to 1 year to permit assessment of long-term morbidity and mortality, with a particular focus on safety and efficacy and renal outcomes.

## Funding

This work was supported by an external collaboration grant from 10.13039/100014468Mallinckrodt Pharmaceuticals.

## Declaration of competing interest

The authors declare the following financial interests/personal relationships which may be considered as potential competing interests: Ethan Weinberg.

• Mallinckodt Pharmaceuticals: study funding, DSMB/Advisory board participation
• BioVie: consulting
• PharmaIN: consulting
• Institute for Medical and Nursing Education: lectures/presentations

Stevan A. Gonzalez.

• Mallinckrodt Pharmaceuticals: advisory board, consulting, Speakers bureau
• Salix Pharmaceuticals: consulting, Speakers bureau
• AbbVie Pharmaceuticals: Speakers bureau

R. Todd Frederick.

• Mallinckrodt Pharmaceuticals: consultant

Raymond A. Rubin.

• Mallinckrodt Pharmaceuticals: clinical trial funding, Speakers bureau

William Tobin.

• Services contract with UPenn to monitor study data

Khurram Jamil.

• Mallinckrodt Pharmaceuticals: employee, patents, stock

Zachary Fricker.

• Mallinckrodt Pharmaceuticals: payments to institution and travel reimbursements

K. Rajender Reddy.

• Grants/contracts: BMS, Intercept, Mallinckrodt, BioVie, Sequana, Grifols, Exact Sciences, HCC-TARGET, NASH-TARGET, Merck
• Royalties/licenses: UpToDate
• Consulting: Spark Therapeutics, Novo Nordisk, Mallinckrodt, Genfit, BioVie
• DSMB/Advisory Board participation: Novartix, Astra Zeneca
• Associate Editor Gastroenterology

No disclosures:

• Suditi Rahematpura, Manhal J. Izzy, Douglass A. Simonetto, Jade Ikahihifo-Bender, Maggie Harte, Grace Kim-Lee, Sherry Witkiewicz
